# The dual role of vulvovaginal candidiasis in HPV infection: implications for vaginal microecology and cervical lesions

**DOI:** 10.3389/fmicb.2025.1724735

**Published:** 2026-01-12

**Authors:** Xiaoyuan Zhang, Mengxing Yao, Yuan Huang, Huiqin Wang, Hui Li, Juanwen Ma

**Affiliations:** Department of Gynecology, Lanzhou Maternal and Child Health Care Hospital, Lanzhou, Gansu, China

**Keywords:** persistent human papillomavirus infection, vaginal microecology, vulvovaginal candidiasis, E6/E7 oncogenes, cervical cancer

## Abstract

Persistent infection with human papillomavirus (HPV) is a major pathogenic factor in the development of cervical lesions and cervical cancer. Its occurrence is not only related to the virus itself but also closely associated with the stability of the host vaginal microecological environment. In particular, microbial dysbiosis caused by vaginal infections such as bacterial vaginosis (BV), trichomoniasis (TV), and vulvovaginal candidiasis (VVC) may facilitate HPV acquisition and persistence. Among these factors, VVC has drawn special attention due to its unique bidirectional role: it may promote persistent HPV infection by inducing local inflammation and disrupting epithelial barrier function, while under certain conditions, it may also activate immune responses that suppress viral activity. This dual nature offers novel mechanistic insights into HPV-related cervical pathogenesis. This review systematically summarizes current evidence on the interplay between persistent HPV infection and vaginal microecological imbalance, with a particular focus on the dual regulatory role of VVC and its potential influence on the expression of the HPV oncogenes E6 and E7 oncogenes. By integrating recent mechanistic findings, the review aims to provide a theoretical foundation and clinical reference for microecology-based interventions to improve HPV-related outcomes and prevent cervical lesions.

## 1 Introduction

The relationship between vaginal microecological dysbiosis and persistent human papillomavirus (HPV) infection, as well as cervical lesions, has attracted growing attention in recent years. Persistent high-risk HPV infection, particularly with types 16 and 18, is a key risk factor for cervical intraepithelial neoplasia (CIN) and cervical cancer (Balasubramaniam et al., 2019). These oncogenic viruses can lead to significant pathological changes in cervical tissues, resulting in a spectrum of lesions ranging from low-grade squamous intraepithelial lesions (LSIL) to high-grade squamous intraepithelial lesions (HSIL) and invasive cervical cancer (Brăila et al., 2025). The interplay between HPV and the vaginal microbiome is complex, as a balanced microbiota is crucial for maintaining vaginal health and preventing infections. Disruptions in this ecosystem, characterized by an overgrowth of pathogenic bacteria or fungi, can create an environment conducive to persistent HPV infections and subsequent cervical lesions (Shen et al., 2024).

The vaginal microbiota is predominantly composed of *Lactobacillus* species, which play a protective role against infections by maintaining an acidic pH and producing antimicrobial substances (France et al., 2022). However, factors such as hormonal changes, sexual behaviors, and antibiotic usage can disrupt this balance and lead to dysbiosis, characterized by a reduction in both the abundance and diversity of Lactobacillus (Chee et al., 2020). Notably, bacterial vaginosis (BV) and vulvovaginal candidiasis (VVC) often coexist with HPV infection, potentially affecting viral persistence and lesion progression (Liu H. M. et al., 2024). Different vaginal infections may exert distinct influences on the course of HPV infection.

Several studies suggest that VVC has a dual role in HPV infection: it may promote viral persistence by inducing inflammation and disrupting the mucosal barrier, but under certain conditions, VVC-induced immune responses can enhance viral clearance. In contrast, BV is consistently linked to a higher prevalence of HPV infection (Long et al., 2023). Mechanistically, vaginal microecological dysbiosis may promote persistent HPV infection and alter E6/E7 oncogene expression, which can disrupt local immune function and cervical epithelial integrity, facilitating virus–host interaction and cervical lesion development (Huang J. et al., 2023; Pagar et al., 2024).

This review focuses on the association between vaginal microecological dysbiosis, persistent HPV infection, and cervical lesions, with particular attention to the potential bidirectional regulatory role of VVC in this process. Building on this, it further examines how imbalances in the vaginal microbiota may influence the expression of HPV oncogenes E6 and E7, and explores microecological intervention strategies—such as the use of probiotics—as potential approaches for the prevention and management of HPV-related cervical lesions. Overall, a deeper understanding of the interplay between HPV infection and the vaginal microbiome will help advance the development of personalized prevention and treatment strategies and improve the scientific basis for cervical health management in women.

## 2 Main body

### 2.1 The relationship between persistent HPV infection and cervical lesions

#### 2.1.1 Classification and distinct pathogenicity of human papillomavirus

High-risk genotypes such as HPV16 and HPV18 are most strongly associated with high-grade CIN and invasive cervical cancer. Persistent high-risk HPV infection drives carcinogenesis through E6 and E7 oncogenes that inactivate tumor suppressor pathways (Wagh et al., 2019).

Persistent infection with HPV16 and HPV18 is strongly linked to high-grade cervical lesions, and co-infection with multiple high-risk types may further increase the risk of progression (Kim et al., 2025; Dickey et al., 2021).

The immune response is crucial in determining HPV infection outcomes; individuals with impaired immunity are more likely to develop persistent infection and cervical lesions (Dom-Chima et al., 2023). Co-infection with pathogens such as *Chlamydia trachomatis* can exacerbate HPV's effects and further increase dysplasia risk (Mbulawa et al., 2022). Together, these factors highlight persistent HPV infection as the central biological prerequisite upon which additional modulators—such as vaginal microecological disturbances—exert their influence on cervical disease development (Leão et al., 2025).

In summary, persistent high-risk HPV infection, together with host immune dysfunction and co-infections, contributes to the development of precancerous and cancerous cervical lesions (Hong et al., 2025).

#### 2.1.2 Mechanisms of persistent HPV infection

Persistent HPV infection is a key driver of cervical cancer (Jain et al., 2023). HPV evades immune detection through multiple strategies, notably via E6 and E7 proteins that disrupt immune surveillance and inactivate tumor suppressor pathways, promoting infected cell survival and the development of precancerous lesions (Zhao et al., 2024; Pan et al., 2022; Chen et al., 2024).

Beyond viral immune evasion, host immunity critically determines HPV infection outcomes. Individuals with impaired immune function, including older women and those with HIV infection, exhibit higher rates of persistent HPV because of reduced T-cell–mediated viral clearance (Tounkara et al., 2021). Co-infections such as Chlamydia trachomatis and other STIs can further impair immunity and promote HPV persistence, reflecting the combined influence of microbial and host factors (Zhao et al., 2023; Lozar and Carchman, 2025).

Overall, persistent HPV infection reflects the interplay between viral immune-evasion mechanisms and host immune competence, forming the biological basis on which vaginal microecological disturbances—including those induced by VVC—may further influence HPV persistence.

#### 2.1.3 The role of HPV oncogenes E6/E7 in carcinogenesis

E6 and E7 are critical drivers of cervical carcinogenesis. They disrupt cell cycle regulation by inactivating the tumor suppressors p53 and Rb, enabling the survival and proliferation of HPV-infected epithelial cells (Hoppe-Seyler et al., 2021; Liu et al., 2022).

Sustained E6/E7 expression is crucial for maintaining malignancy in HPV-positive cervical cancer. Their continuous activity supports cancer cell survival and proliferation, highlighting E6/E7 as essential biomarkers of disease progression (Liu et al., 2022; Inturi and Jemth, 2021). Moreover, E6 and E7 expression is tightly regulated by multiple factors, including the cellular environment and interactions with viral or host elements (Schichl and Doorbar, 2025). These regulatory influences are particularly relevant when considering how vaginal microecological disturbances—such as those caused by VVC—may indirectly affect HPV oncogene activity.

In summary, E6 and E7 promote HPV-driven carcinogenesis by sustaining the proliferation and survival of infected cells, forming a critical mechanistic basis for understanding factors—including VVC-related dysbiosis—that may modulate HPV persistence and progression (Hoppe-Seyler et al., 2021; Liu et al., 2022).

### 2.2 Composition and function of the vaginal microecological environment

#### 2.2.1 Composition of normal vaginal microecology

The vaginal microecology, dominated by Lactobacillus species such as *L. crispatus, L. gasseri*, and *L. iners*, maintains a protective environment through acid production and antimicrobial activity that suppress pathogenic overgrowth and support local immune homeostasis (Deka et al., 2021). Recent high-throughput sequencing studies have shown that the vaginal microbiota can be classified into community state types (CSTs), with *Lactobacillus-dominant* CSTs (I, II, III, and V) being associated with low inflammation and reduced susceptibility to persistent HPV infection (Musa et al., 2023; Zhang Y. et al., 2024). In contrast, CST IV—characterized by reduced Lactobacillus abundance and increased microbial diversity—has been consistently linked to epithelial barrier impairment, heightened inflammatory cytokines, and increased risk of persistent high-risk HPV infection (Zhang Y. et al., 2024; Lin et al., 2022; Shen et al., 2022).

Maintaining vaginal microbiome balance is vital for sustaining epithelial barrier integrity. Loss of *Lactobacillus* dominance predisposes women to dysbiosis, including BV and VVC, and may alter mucosal immunity in ways relevant to HPV persistence (Huang J. L. et al., 2023). Understanding these CST-based ecological patterns is essential for interpreting how VVC-associated disruptions of normocenosis may facilitate HPV persistence through inflammation, immune modulation, and loss of Lactobacillus-mediated antiviral mechanisms (Laghi et al., 2021; Garmendia et al., 2024).

In summary, *Lactobacillus-dominant* communities therefore serve as a key protective ecological state against HPV persistence, providing the foundational microenvironment on which VVC-induced dysbiosis may exert downstream effects on HPV-related disease (Laghi et al., 2021; Garmendia et al., 2024).

#### 2.2.2 Manifestations and diagnosis of vaginal microbial dysbiosis

Vaginal microbial dysbiosis, marked by an imbalance in the microbiota, often manifests as abnormal discharge linked to BV or VVC. Symptoms such as abnormal discharge and irritation reflect alterations in microbial composition and mucosal inflammation.

Diagnosis of vaginal microbial dysbiosis combines clinical assessment and laboratory testing. Amsel's criteria and the Nugent score are commonly used, with the latter—based on Gram-stained smears—serving as the gold standard; scores ≥7 indicate reduced Lactobacillus and increased anaerobes. Vaginal pH >4.5 also suggests dysbiosis. Molecular methods such as 16S rRNA sequencing now provide finer-resolution profiling of the vaginal microbiota, supporting more precise characterization of dysbiosis relevant to HPV and VVC interactions (Bennett et al., 2020; Li et al., 2025; Wei and Chen, 2021). Beyond sequencing-based approaches, fluorescence *in situ* hybridization (FISH) has become a useful complementary tool for identifying biofilm-associated BV. Building on the work of Swidsinski's group, recent studies show that FISH can directly visualize dense *Gardnerella* biofilms on the vaginal epithelium—structural details not captured by Gram staining or 16S profiling (Swidsinski et al., 2023). These findings reinforce BV as a biofilm-driven condition and help clarify its link to persistent HPV infection.

In summary, the diagnosis of dysbiosis provides essential context for understanding how VVC-related microbial shifts may alter susceptibility to HPV acquisition and persistence (Łaniewski et al., 2024; Mazinani et al., 2025; Lee et al., 2022).

### 2.3 The relationship between vaginal microecological disorder and HPV infection

#### 2.3.1 BV and HPV infection

BV is a major disruption of the vaginal microbiome, marked by reduced *Lactobacillus* and overgrowth of anaerobes, and is associated with greater susceptibility to high-risk HPV infection. Beyond this compositional shift, BV also features a dense *Gardnerella*-dominated biofilm adherent to the vaginal epithelium, which protects resident bacteria from immune clearance and sustains chronic low-grade inflammation. This biofilm structure provides protection against host immune clearance and maintains chronic low-grade inflammation, further weakening epithelial tight junctions and amplifying HPV entry opportunities (Shang et al., 2024; Ma et al., 2022). It disrupts the vaginal ecosystem and immune balance, creating inflammation that facilitates HPV entry and persistence, while impaired epithelial integrity and altered immunity further increase infection risk and hinder viral clearance (Long et al., 2023; Martins et al., 2023). Furthermore, inflammatory cytokines released during BV can promote HPV replication and persistence, contributing to the progression of cervical lesions over time.

Statistical data from various studies corroborate the association between BV and increased HR-HPV infection rates. A meta-analysis has shown women with BV are 2.68 times more likely to have HR-HPV (Martins et al., 2023), and a study of 14,679 women found BV is more prevalent in those with cytological abnormalities, suggesting it may also be linked to lesion severity (Long et al., 2023). Additional research has highlighted that the prevalence of BV is notably higher in women with CIN, further establishing BV as a potential co-factor in HPV-related pathogenesis (Zhou et al., 2023). These findings highlight the need to screen for BV in women with HPV, as treating BV may help reduce HPV persistence and the risk of cervical disease progression.

In summary, BV increases susceptibility to HPV and may worsen HPV-related clinical outcomes. Understanding this interaction is vital for developing strategies to prevent and treat cervical cancer, especially in high-risk populations. Targeted management of BV could help reduce HPV persistence and disease progression, underscoring the importance of integrated women's health approaches that consider the vaginal microbiome's role in HPV infection.

#### 2.3.2 Trichomonas vaginalis (TV) and HPV infection

TV is the most common non-viral sexually transmitted infection globally and causes marked disruption of the vaginal microenvironment, promoting HPV persistence. TV infection also produces direct epithelial microabrasions and disrupts tight-junction proteins, creating physical entry portals for HPV (Hinderfeld et al., 2019). In addition, parasite-secreted proteases intensify mucosal inflammation and impair epithelial repair, thereby enhancing HPV's ability to establish persistent infection (Zimmann et al., 2022). It reduces beneficial *Lactobacillus* populations, impairs local immunity, and creates conditions that enhance pathogen survival and increase susceptibility to HPV infection (Heikal et al., 2023). The inflammatory response to TV increases epithelial HPV receptor expression and also upregulates CD151 and HSPG2, which mediate viral entry (Sheng et al., 2025). Additionally, inflammatory cytokines induced by *Trichomonas vaginalis* infection create a microenvironment that promotes HPV replication and persistence, increasing the risk of CIN and cervical cancer (Hamar et al., 2023).

Moreover, co-infection with *Trichomonas vaginalis* and HPV exacerbates cervical lesion severity, with studies showing a higher prevalence of HSIL in women harboring both infections than in those with HPV alone (Idrak et al., 2024). Thus, *Trichomonas vaginalis* facilitates HPV infection and lesion progression through immune evasion and inflammation. The interaction between TV and HPV highlights the need for comprehensive STI screening and management to reduce cervical cancer risk. Given the high co-infection rates, further studies are warranted to clarify their molecular interplay and guide targeted therapies to improve outcomes in affected women (Hamar et al., 2023).

In conclusion, the complex relationship between Trichomonas vaginalis infection and HPV persistence underscores the need for integrated prevention and treatment strategies.

#### 2.3.3 VVC and HPV infection: a dual perspective

Vulvovaginal candidiasis (VVC), mainly caused by *Candida albicans albicans*, significantly influences HPV infection dynamics. *Candida albicans* overgrowth disrupts the vaginal microenvironment and induces inflammation that may promote HPV persistence. A defining pathogenic feature of VVC is the release of *Candida albicans*lysin, a pore-forming toxin that induces epithelial cell lysis and stimulates a strong IL-1β-dominated inflammatory response (Valentine et al., 2024; Cheng K. O. et al., 2024). This mucosal damage increases exposure of basal keratinocytes—the primary HPV target cells—thereby promoting viral persistence under conditions of dysbiosis. Studies have shown that women with concurrent VVC have a higher likelihood of persistent HPV infection, suggesting that the inflammation caused by fungal infection creates conditions favorable for viral survival (Chen et al., 2025). Furthermore, *Candida albicans* overgrowth disrupts *Lactobacillus* dominance, weakening natural defenses against HPV and increasing viral load and cervical lesion risk (Li et al., 2024b). Additionally, the inflammatory cytokines released during VVC further enhance HPV immune evasion, promoting persistence. This dual effect highlights the complex interaction between fungal and viral pathogens and underscores the need to better understand how VVC contributes to HPV-related cervical disease.

Conversely, *Candida albicans* may also inhibit HPV activity and protect against initial infection under certain conditions. A cross-sectional study found that women with VVC had a lower likelihood of concurrent HPV detection (Gao et al., 2021), possibly due to immune responses induced by *Candida albicans* that enhance local defenses or through antifungal metabolites that alter the vaginal environment to hinder viral establishment. These effects may vary with host health, hormonal status, and microbiota composition. Thus, while VVC can promote HPV persistence via inflammation, it may also offer transient protection against infection, underscoring the complex, context-dependent nature of VVC–HPV interactions and the need for personalized management strategies.

### 2.4 The dual role mechanism of VVC and HPV infection

#### 2.4.1 Mechanisms by which VVC promotes persistent HPV infection

VVC, primarily caused by *Candida albicans albicans*, is increasingly recognized as a factor influencing HPV infection dynamics. It promotes persistent HPV infection by inducing local inflammation and immune modulation. *Candida albicans* triggers immune cell recruitment, disrupting the epithelial barrier and enhancing HPV entry and persistence. Inflammatory cytokines released during infection can alter receptor expression and co-factors essential for HPV replication, while changes in vaginal pH and microbiome composition further create a favorable environment for viral survival (Long et al., 2023).

Beyond inflammation, virulence factors produced by *C. albicans*—including secreted aspartyl proteases (SAPs) and *Candida albicans*lysin—directly damage epithelial tight junctions (e.g., E-cadherin, occludin), increasing the exposure of basal keratinocytes, the primary targets of HPV infection (Bras et al., 2024; Kumar et al., 2022). These structural disruptions facilitate viral access to deeper epithelial layers and promote persistent HPV colonization. VVC-associated dysbiosis with reduced Lactobacillus dominance can weaken lactic-acid–mediated antiviral defense and increase vaginal pH, forming a microenvironment that supports HPV persistence (Bautista et al., 2025). These microecological shifts are consistent with clinical evidence that women with chronic *C. albicans* infection exhibit higher rates of persistent rather than incident HPV infection (Chen et al., 2025).

An additional mechanism linking VVC to HPV persistence is the formation of *Candida albicans* biofilms. Biofilm-embedded *C. albicans* displays enhanced resistance to immune clearance and antifungal therapy, leading to chronic mucosal inflammation (Yan et al., 2025; David and Solomon, 2023). Such persistent inflammation—frequently observed in recurrent VVC—impairs epithelial repair, maintains elevated cytokines, and sustains dysbiosis (Yan et al., 2025). These biofilm-driven alterations favor a shift toward CST IV–like microbiota with reduced Lactobacillus dominance, an ecological profile strongly associated with persistent high-risk HPV infection (Mei et al., 2022). Although direct clinical evidence remains limited, biofilm-associated immune modulation and barrier disruption provide a plausible pathway by which VVC may facilitate prolonged HPV survival.

Furthermore, VVC-induced inflammation may potentiate HPV oncogenic activity. Pro-inflammatory cytokines (e.g., IL-1β, IL-6, TNF-α) activated via NF-κB and JAK/STAT signaling during *C. albicans* infection have been shown to enhance HPV long control region (LCR) promoter activity, leading to increased E6/E7 transcription (Williams et al., 2011). Oxidative stress generated in inflamed mucosa further augments HPV early gene expression and may promote viral DNA integration. These mechanisms provide the biological basis for subsequent discussions on how VVC-related inflammation may act as an upstream regulator of HPV E6/E7 expression.

In summary, VVC promotes persistent HPV infection through epithelial barrier disruption, inflammatory cytokine activation, oxidative stress, and microecological imbalance, all of which create a permissive environment for HPV survival and oncogene expression. Understanding these mechanisms is essential for developing targeted therapies to restore vaginal microbiota balance and reduce HPV-related risks.

#### 2.4.2 Potential mechanisms of VVC in suppressing HPV infection

VVC exhibits a dual role in HPV infection, although the evidence for a suppressive effect remains much weaker than that for its promoting role. It stimulates innate and adaptive immunity, recruiting macrophages and neutrophils that release cytokines to clear fungi and create conditions less favorable for HPV persistence. *Candida albicans*-induced activation of Toll-like receptors enhances IFN-γ and TNF-α expression, promoting antiviral defense and, in some cases, reducing HPV load. However, these findings are based on limited and largely cross-sectional evidence, and their reproducibility across populations remains unclear. This effect is context-dependent, influenced by co-infections and host immune status. While VVC may confer transient and modest protection, other infections such as BV show a stronger association with HPV, suggesting that VVC's protective role is minor, inconsistent, and not comparable in strength to its HPV-promoting mechanisms (Long et al., 2023; Wang et al., 2020).

Interactions between *Candida albicans* and other vaginal microorganisms critically influence HPV dynamics. Under normal conditions, *Lactobacillus* species maintain microbial balance and protect against pathogens, including HPV. During VVC, *Candida albicans* overgrowth disrupts this balance, creating a dysbiotic state that may favor HPV and other pathogens. Although some ecological interactions could theoretically reduce the abundance of certain bacteria, such effects have not been shown to meaningfully suppress HPV activity. Instead, VVC-associated increases in pH and microenvironmental changes can promote HPV survival and replication. Given the imbalance in evidence, the HPV-suppressive effect of VVC should be regarded as hypothetical and secondary to its well-established role in supporting HPV persistence (Li et al., 2024b; Fu and Zhang, 2023).

In conclusion, although VVC-related immune activation may exert limited inhibitory effects on HPV under certain conditions, the current evidence is insufficient to place this mechanism on equal footing with the extensively documented pathways through which VVC promotes HPV persistence. Further longitudinal and mechanistic studies are required to clarify the true extent and significance of this proposed suppressive role.

#### 2.4.3 Host factors influencing the interaction between VVC and HPV

The interplay between vaginal microbiota—particularly VVC—and HPV infection is strongly shaped by host immune and hormonal factors. A healthy, *Lactobacillus-dominant* microbiota supports robust immune responses that facilitate HPV clearance, whereas dysbiosis in VVC can weaken local defenses, promoting viral persistence and cervical lesion development (Kazlauskaite et al., 2025). Hormonal fluctuations from menstruation, pregnancy, or contraceptive use further influence microbiome composition and immunity; while estrogen favors Lactobacillus growth, other hormonal shifts may increase susceptibility to both VVC and HPV (Cui et al., 2025). These combined effects create conditions that can sustain HPV infection and drive progression toward cervical dysplasia and cancer.

Individual factors, including genetic predisposition and lifestyle, further influence the dual role of VVC in HPV interactions. Variations in immune-related genes can alter host responses to HPV, with certain polymorphisms linked to increased risk of persistent infection and cervical lesion development (Na et al., 2025). Additionally, lifestyle factors, including smoking, diet, and sexual behavior, affect the vaginal microbiome and immunity, and smoking is a notable risk factor for persistent HPV, likely via immunosuppression that worsens dysbiosis and HPV-related complications (Zukiene et al., 2025). Furthermore, individual variations in vaginal microbiota, shaped by factors such as sexual activity and hygiene practices, also affect susceptibility to VVC and HPV co-infections. These differences underscore the need for personalized approaches to understanding host–microbiome interactions, which could guide targeted prevention and treatment strategies for cervical cancer (Ventura et al., 2024).

In summary, the relationship between VVC and HPV is shaped by host factors such as immune status, hormonal balance, and individual variability. Understanding these influences is crucial for reducing the risks of HPV persistence and cervical lesions, especially in women with VVC. Future research should clarify how host factors interact with the vaginal microbiome and HPV to inform personalized prevention and treatment strategies that enhance women's reproductive health.

### 2.5 The association between HPV oncogenes E6/E7 expression and vaginal microecological disorder

#### 2.5.1 E6/E7 gene expression and correlation with HR-HPV infection

E6 and E7 expression is central to cervical carcinogenesis, as E6 degrades p53 and E7 inactivates pRb, disrupting cell cycle control and promoting uncontrolled proliferation. Elevated E6/E7 mRNA levels serve as biomarkers of HPV oncogenic potential. In a study of 1,327 women, those with HR-HPV showed significantly higher E6/E7 expression than those with low-risk types. Increased E6/E7 levels correlate with precancerous lesions and cervical cancer, while BV may act as an independent HR-HPV risk factor by inducing microecological changes that enhance E6/E7 expression (Huang J. et al., 2023).

Beyond cervical carcinogenesis, E6/E7 mRNA expression is also implicated in other HR-HPV–related malignancies, including head and neck cancers. Expression patterns vary by HPV genotype, with HPV16 and HPV18 showing the highest oncogenic potential. Mutations within E6 and E7 can alter expression levels and influence cancer aggressiveness; for instance, specific E7 variants are linked to increased risk of high-grade cervical lesions (Evande et al., 2023). Moreover, E6/E7 mRNA detection offers greater sensitivity and specificity for identifying HSIL than traditional HPV DNA testing, highlighting its value in clinical diagnostics (Evande et al., 2023; Plisko et al., 2024).

The regulation of E6/E7 expression is intricately linked to host cellular pathways and the tumor microenvironment. E6 and E7 can modulate the immune landscape, fostering tumor progression and immune evasion (Pandey et al., 2025). Additionally, their interactions with key signaling pathways, such as Wnt/β-catenin, further contribute to HPV-driven tumorigenesis (Donmez et al., 2022).

In conclusion, E6 and E7 mRNA expression serves as a key indicator of HR-HPV carcinogenic potential, closely correlating with cervical cancer progression and high-grade lesions. Incorporating E6/E7 mRNA testing into clinical practice could enhance early detection and risk assessment. Further elucidation of the regulatory mechanisms controlling their expression may guide the development of targeted therapies to mitigate HR-HPV–driven carcinogenesis.

#### 2.5.2 The impact of dysbiosis on E6/E7 expression

The relationship between vaginal dysbiosis and HPV oncogene expression is key to understanding microbial influences on cervical carcinogenesis. Dysbiosis—marked by reduced *Lactobacillus* and increased pathogenic bacteria—creates conditions favorable for persistent HR-HPV infection. In HPV-16–transformed SiHa cells, specific *Lactobacillus* species, particularly *L. crispatus* and *L. gasseri*, were shown to modulate and suppress basal E6/E7 expression and oncoprotein production, highlighting their potential protective role against HPV-driven malignancy (Nicolò et al., 2023). In contrast, dysbiosis-associated bacteria such as *Gardnerella vaginalis* and *Megasphaera micronuciformis* enhance E6/E7 expression and oncoprotein production. *M. micronuciformis* further decreases p53 and pRb levels, promoting S-phase progression and potential malignant transformation of cervical epithelial cells. These findings illustrate the dual role of the vaginal microbiota—*Lactobacillus* species mitigate oncogenic risk, whereas dysbiotic bacteria exacerbate it. Maintaining a healthy, *Lactobacillus-dominant* microbiome may thus help prevent HPV-related cervical lesions, and probiotic interventions show promise in counteracting dysbiosis-induced upregulation of E6/E7 expression and associated cancer risk (Bi et al., 2023). Thus, understanding how specific bacterial species regulate HPV oncogene expression is crucial for developing targeted therapies and preventive strategies against HPV-related cervical cancer.

#### 2.5.3 Influence of VVC and *Candida albicans albicans* on HPV E6/E7 expression

Recent evidence indicates that *Candida albicans albicans*–induced inflammation and epithelial injury may modulate HPV oncogene expression, offering a mechanistic link between VVC and cervical carcinogenesis. *C. albicans* stimulates strong IL-1β, IL-6, and TNF-α responses via NF-κB and JAK/STAT activation, generating an inflammatory milieu that can enhance HPV early promoter activity and upregulate E6/E7 transcription (Moyes et al., 2015; Valand and Girija, 2021).

Moreover, *Candida albicans* secretes aspartyl proteases (SAPs) that disrupt epithelial tight junctions and compromise barrier integrity, increasing exposure of basal keratinocytes—the primary HPV target cells—and potentially facilitating early viral gene expression, including E6/E7 (Bras et al., 2024). Metabolic byproducts such as ethanol and short-chain organic acids further alter vaginal pH and oxidative stress, factors known to affect HPV replication and early gene transcription (Hickey et al., 2025).

Conversely, acute VVC can activate strong innate immune responses, particularly TLR2/TLR4 pathways and IFN-γ production, which may exert antiviral effects and suppress E6/E7 mRNA expression. This bidirectional influence aligns with epidemiologic findings showing that VVC may either promote HPV persistence or, in certain contexts, reduce HPV detectability through enhanced immune clearance (Chen et al., 2025; Miró et al., 2017).

#### 2.5.4 Application value of E6/E7 gene testing in clinical diagnosis

E6/E7 gene testing has become a key tool in the clinical diagnosis of HPV infections, especially in cervical cancer. E6/E7 mRNA testing demonstrates high specificity and sensitivity for HR-HPV associated with CIN and cervical cancer, outperforming HPV DNA testing in predicting high-grade lesions (Pankaj et al., 2024). The detection of E6/E7 mRNA also reflects active viral replication and oncogenic potential, making it essential for evaluating disease progression risk in HPV-infected patients (He et al., 2021).

However, E6/E7 gene testing has several limitations. False positives can occur, particularly in low HPV prevalence settings, leading to unnecessary anxiety and procedures such as colposcopy or biopsy (Zhang et al., 2023). Additionally, the interpretation of E6/E7 results can be complicated by the presence of multiple HPV types, as co-infections may influence the expression levels of these oncogenes (Dust et al., 2022). Moreover, factors such as immune status and comorbidities can affect test accuracy and influence cervical cancer risk assessment (Kotian et al., 2024).

In conclusion, E6/E7 gene testing shows great potential as an auxiliary diagnostic tool for HPV-related diseases, but its benefits must be weighed against inherent limitations. Continued advances in molecular diagnostics are essential to improve its accuracy and clinical utility. Integrating E6/E7 testing into routine practice—supported by patient education and counseling—will help maximize its diagnostic value while minimizing potential drawbacks, ultimately improving outcomes in HPV management.

### 2.6 Clinical intervention of vaginal microecological repair and HPV infection

#### 2.6.1 Microecological regulation treatment strategies

Probiotic therapy has gained attention as a strategy to restore vaginal microecology, particularly for BV and VVC. *Lactobacillus* strains maintain microbiota balance by producing lactic acid, lowering pH, and inhibiting pathogens such as *Gardnerella* and *Atopobium* (Han et al., 2025; Jawanda et al., 2024). Clinical trials, including those using *Lactobacillus crispatus*, have shown that probiotics can restore vaginal flora, reduce Nugent scores, and increase *Lactobacillus* abundance, improving outcomes in recurrent infections (Jawanda et al., 2024). By enhancing anti-inflammatory cytokines and suppressing pro-inflammatory pathways, probiotics reduce inflammation and may strengthen mucosal immunity, potentially lowering HPV persistence and cervical lesion risk (Han et al., 2025; Jawanda et al., 2024; Schuster et al., 2024). Overall, integrating probiotics into treatment protocols offers a promising approach to restoring vaginal health and preventing recurrence.

Antibiotics and antifungal agents remain central to managing vaginal infections but can disrupt the vaginal microbiota balance. Drugs such as metronidazole and clindamycin effectively treat BV yet often deplete beneficial *Lactobacillus*, leading to dysbiosis and frequent relapse (Schuster et al., 2024; Muzny and Sobel, 2022). Similarly, antifungals clear *Candida albicans* infections but may alter microbial composition, foster resistance, and contribute to recurrence (Bradfield Strydom et al., 2023). Combining antibiotics with probiotics may mitigate antibiotic-induced microbiota disruption, improve efficacy, and reduce recurrence while preserving beneficial microbes essential for vaginal health (Han et al., 2025; Jawanda et al., 2024).

#### 2.6.2 The impact of microecological restoration on HPV clearance rates

Recent research has highlighted the pivotal role of vaginal microbiota in HPV infection and clearance. A *Lactobacillus-dominant* microbiome is associated with reduced risk of persistent HPV and cervical lesions. In a pilot study of 100 women, those with persistent HR-HPV had lower *Lactobacillus* and higher pathogenic bacteria than those who cleared the virus (Mei et al., 2022). A longitudinal study further showed that higher baseline *Lactobacillus iners* abundance correlated with reduced HPV clearance within 1 year (Shi et al., 2022). These findings emphasize that vaginal microbiota composition influences immune responses and viral persistence, suggesting that restoring a healthy microbiome may promote HPV clearance.

Microecological restoration, especially through probiotic therapy, shows promise in enhancing HPV clearance. In a controlled study, intravaginal *Lactobacillus crispatus* significantly reduced HPV viral load and improved clearance compared with placebo (Liu Y. et al., 2024). These findings suggest that probiotics may restore the vaginal microbiota and enhance local immunity, promoting HPV clearance; a systematic review further confirmed that *Lactobacillus* dominance is linked to lower HPV persistence and cervical dysplasia rates (Pai et al., 2025).

Moreover, the mechanisms by which microecological restoration aids in HPV clearance are becoming clearer. *Lactobacillus* can increase antimicrobial substance production, modulate immunity, reduce inflammation, and improve epithelial barrier integrity, collectively creating conditions unfavorable for HPV persistence (Papamentzelopoulou and Pitiriga, 2025). In this context, the role of vaginal probiotics is not merely to restore microbial balance but also to enhance the host's immunological defenses against viral infections.

In conclusion, restoring vaginal microecology through probiotics and related interventions shows strong potential for enhancing HPV clearance. Clinical evidence indicates that a *Lactobacillus-dominant* microbiome is associated with reduced HPV persistence and fewer cervical lesions. As understanding of vaginal microbiota–HPV interactions deepens, microecological restoration may become an effective strategy for reducing cervical cancer risk. Further large-scale studies are needed to confirm these findings and refine microbiota-based prevention and treatment approaches.

#### 2.6.3 The significance of VVC treatment in HPV infection management

Treating VVC is important for supporting HPV infection management. As a common fungal infection, VVC can disrupt vaginal microecology and alter the dynamics of HPV persistence and progression (Dovnik et al., 2015). Although VVC does not significantly increase the risk of HPV-related cytological abnormalities, its treatment helps restore vaginal microecology, strengthen local immunity, thereby improving the host environment related to HPV clearance rather than directly modifying lesion risk (Long et al., 2023). The interplay between VVC and other infections such as BV underscores the need for comprehensive vaginal health management; notably, BV may protect against cytological abnormalities in HPV-infected women, highlighting the importance of a balanced and *Lactobacillus-dominant* microbiome in modulating HPV persistence (Holali Ameyapoh et al., 2021). Thus, treating VVC not only relieves symptoms but also contributes to overall microecological stability, which is relevant to broader HPV management strategies.

Preventive strategies should integrate VVC management with HPV screening, vaccination, and probiotic-based microbiome modulation to maintain vaginal health and reduce HPV risk (Brennan et al., 2024). Addressing VVC and other infections can improve outcomes in women susceptible to HPV-related complications. Education on sexual health further supports prevention. Overall, effective VVC treatment is a key component of HPV management by supporting microecological and immunological homeostasis, highlighting the need for continued research into vaginal microecology–HPV interactions.

### 2.7 Microecological-viral interaction model in the development of cervical cancer

#### 2.7.1 Pathways by which microbial dysbiosis promotes cervical cancer development

Microbial dysbiosis in the female reproductive tract contributes to cervical cancer through chronic inflammation and immune suppression (Huang et al., 2024). A *Lactobacillus-dominant* microbiome protects against infection, while reduced *Lactobacilli* and increased pathogens are linked to persistent HPV infection (Huang et al., 2024). Dysbiosis disrupts immune balance and elevates IL-6 and TNF-α levels, creating a pro-inflammatory, oncogenic microenvironment (Shen et al., 2024).

Moreover, immune suppression caused by dysbiosis further hinders HPV clearance. A dysbiotic microbiome alters local immunity, promoting recruitment of regulatory T cells and myeloid-derived suppressor cells that weaken antiviral responses (Qingqing et al., 2021). This immune evasion enables HPV persistence and progression to CIN and cervical cancer. Thus, maintaining a balanced vaginal microbiome is essential for preventing HPV-related malignancy.

Dysbiosis and chronic inflammation form a self-reinforcing cycle that hinders HPV management, highlighting the need for microbiota-restoring therapies. Probiotics can rebalance the microbiome, reduce inflammation, and promote HPV clearance, potentially lowering cervical cancer risk (Mazinani et al., 2025). Integrating microbiome analysis into clinical practice may enable personalized prevention and treatment strategies targeting both microbial imbalance and inflammation.

#### 2.7.2 HPV virus and microbiome's synergistic carcinogenic mechanism

The interaction between HPV and the vaginal microbiome plays a key role in cervical carcinogenesis. Certain microbial communities influence cellular signaling pathways exploited by high-risk HPV types such as HPV-16 and HPV-18, which integrate into the host genome and upregulate E6/E7, disrupting p53 and Rb pathways (Sims et al., 2021). Reduced *Lactobacillus* dominance increases HPV persistence, while anaerobes like *Gardnerella* and *Prevotella* induce inflammation that promotes viral integration and tumor development (Nieves-Ramírez et al., 2021; Lebeau et al., 2022).

Furthermore, dysbiosis of the vaginal microbiome alters local immune responses and weakens host defenses against HPV. Reduced *Lactobacillus* and increased pathogenic bacteria elevate inflammatory cytokines such as IL-6 and TNF-α, which are associated with CIN progression, suggesting that chronic inflammation synergizes with HPV in driving carcinogenesis (Li X. et al., 2024; Leon-Gomez and Romero, 2024; Kamzayeva et al., 2025).

Moreover, HPV can also modify the vaginal microbiome, creating a feedback loop that worsens dysbiosis and enhances oncogenic potential. The E7 oncoprotein downregulates antimicrobial peptides essential for microbial balance, enabling pathogenic bacteria to proliferate (Lebeau et al., 2022). This shift not only facilitates HPV persistence but also promotes pro-inflammatory mediator expression, further driving carcinogenesis.

In conclusion, the interplay between HPV and the vaginal microbiome synergistically regulates cellular signaling pathways that drive cervical carcinogenesis. Understanding this relationship may enable the development of therapeutic strategies—such as probiotic interventions—to restore microbial balance, strengthen host defenses, and reduce cervical cancer risk (Uysal et al., 2022; Gutierrez Salmean et al., 2024). Future research should identify microbial signatures linked to HPV persistence and clarify how these microorganisms influence oncogenic signaling mechanisms.

#### 2.7.3 Latest theoretical models and future research directions

The interplay between the vaginal microbiome and HPV infection has gained increasing attention, especially with the use of multi-omics technologies that enable a deeper understanding of their interactions. By integrating genomics, transcriptomics, proteomics, and metabolomics, multi-omics approaches offer a comprehensive view of vaginal microbial communities and their functions in HPV infection. A *Lactobacillus*-dominated microbiome is essential for maintaining homeostasis and protecting against HPV and other infections (Kyrgiou and Moscicki, 2022). Dysbiosis, marked by reduced *Lactobacillus* and increased pathogens such as *Gardnerella vaginalis*, is linked to greater susceptibility to HPV infection and cervical lesion progression (Zukiene et al., 2025; Ye and Vaginal, 2024). Shotgun metagenomic studies reveal that HPV-associated microbiome changes extend beyond composition, involving alterations in metabolic pathways, suggesting that specific microbial taxa may drive HPV persistence and CIN development (Yang et al., 2025; Valentine et al., 2025).

HPV and the vaginal microbiome interact bidirectionally, with HPV worsening dysbiosis in a self-perpetuating cycle that promotes cervical disease (Li M. et al., 2024). Elucidating the molecular mechanisms underlying this interaction—such as how bacterial metabolites affect HPV gene expression and immune responses—may reveal therapeutic targets. Additionally, identifying microbial biomarkers linked to HPV persistence and lesion progression could also enable novel diagnostic and preventive strategies (Shen et al., 2024; Yang et al., 2024).

As research advances, it is crucial to examine these interactions across diverse populations, given that vaginal microbiome composition varies with ethnicity, age, and environmental factors (Mbabazi et al., 2025). Future studies should also investigate how host genetics and immune responses shape the microbiome–HPV relationship. A comprehensive, personalized approach—integrating microbiome profiling, immune status, and multi-omics data with clinical outcomes—will be key to developing precision strategies for HPV-related disease management and improving prevention and treatment of cervical cancer.

### 2.8 Future research hotspots and challenges

#### 2.8.1 Development prospects of precision microbiota regulation technologies

Precision microbiota regulation technologies, particularly microbiome editing, hold promise for managing HPV infection and cervical lesions. Using CRISPR-Cas9, synthetic biology, and advanced sequencing, microbiome editing enables targeted modification of microbial genes and functions to restore balance and enhance host health. Specific *Lactobacillus* species have been shown to protect against HPV persistence and cervical cancer by modulating local immunity and maintaining vaginal homeostasis (Papamentzelopoulou and Pitiriga, 2025). Microbiome editing could increase beneficial bacteria, reduce harmful species, and create a microenvironment that strengthens immune defense against HPV.

Beyond gynecological health, microbiome editing holds promise for broader clinical applications. The gut microbiota plays a key role in systemic diseases such as obesity, diabetes, and inflammatory bowel disease, highlighting the value of targeted microbial modulation for therapeutic benefit (Yang et al., 2025). Strategies such as dietary intervention, probiotic supplementation, and fecal microbiota transplantation have demonstrated potential to improve metabolic function and reduce inflammation (Wang et al., 2025). Integrating precision microbiota regulation into clinical practice could enable treatments tailored to individual microbiome profiles, improving efficacy and reducing adverse effects.

Despite its promise, microbiome editing faces several challenges. Ethical concerns about genetic modification, the need for comprehensive safety evaluations, and the complexity of host–microbiome interactions hinder clinical translation. Individual variability in microbiome composition also demands deeper insight into the mechanisms linking microbiota to health and disease. Future research should clarify these mechanisms, refine editing technologies, and establish clinical standards to ensure the safe and effective implementation of microbiome editing in healthcare.

In conclusion, precision microbiota regulation technologies, especially microbiome editing, offer a transformative approach to managing dysbiosis-related and infectious diseases such as HPV. These advanced tools enable personalized interventions that restore microbial balance and improve overall patient health. Continued interdisciplinary research and collaboration will be vital to fully realize the clinical potential of microbiome editing and develop innovative solutions for complex health challenges.

#### 2.8.2 Investigating the molecular mechanisms of HPV infection and microbiota interaction: key molecular targets and signaling pathways

The complex interplay between HPV infection and the vaginal microbiota is now recognized as a key factor in cervical lesion and cancer development. Recent studies have identified molecular targets and signaling pathways underlying this interaction. Vaginal dysbiosis—marked by reduced *Lactobacillus* and increased *Gardnerella* and *Prevotella*—is linked to persistent HPV infection and CIN progression (Cheng et al., 2020; Chen et al., 2021). These microbial shifts elevate IL-6 and TNF-α levels, inducing chronic inflammation and creating a microenvironment that supports HPV persistence (Qingqing et al., 2021; Leon-Gomez and Romero, 2024). Moreover, key signaling pathways such as NF-κB and JAK/STAT play central roles in mediating inflammation and facilitating HPV immune evasion, thereby contributing to viral persistence and disease progression (Kazlauskaite et al., 2025; Zhang Z. et al., 2024).

Furthermore, the interaction between HPV and the vaginal microbiota may also regulate the expression of viral oncogenes E6 and E7, which drive cellular transformation and cancer progression (Uysal et al., 2022; Fang et al., 2022). Dysbiotic microbiota can alter the local immune microenvironment by impairing antigen-presenting cells, such as Langerhans cells, reducing the host's ability to clear HPV (Dai et al., 2022). Microbiota-targeted therapies, including probiotics, may help restore microbial balance and enhance immune defenses against HPV (Dahlstrom et al., 2023; Cheng L. et al., 2024).

In summary, the interaction between HPV and the vaginal microbiota involves a complex network of microbial dysbiosis, immune modulation, and signaling pathways that promote HPV persistence and cervical lesion development. Elucidating these molecular mechanisms could inform novel therapeutic strategies for preventing HPV-related diseases and improving women's health. Future research should aim to identify specific microbial biomarkers and their functional roles in HPV infection, enabling personalized approaches to cervical cancer prevention and management.

#### 2.8.3 The necessity of large-scale clinical cohort studies

Investigating the relationship between vaginal microbiota, HPV persistence, and cervical lesions requires large, multi-center longitudinal cohort studies encompassing diverse populations. Such designs enhance the generalizability of findings, as vaginal microbiota composition varies with genetic, environmental, and lifestyle factors. Evidence indicates that *Lactobacillus-dominant* microbiota confers protection against HPV persistence, whereas a shift toward a diverse, dysbiotic community increases the risk of cervical lesions (Kazlauskaite et al., 2025). Multi-center longitudinal studies enable the capture of diverse microbiota profiles and HPV genotypes, allowing researchers to track temporal changes in microbiota–HPV interactions, infection dynamics, and lesion progression. For instance, longitudinal analyses have shown that *Lactobacillus*-dominated community state types are linked to higher HPV clearance rates (Shi et al., 2022). This temporal approach is essential for understanding how microbiota shifts influence HPV persistence and CIN development. Including diverse demographic samples can also help identify microbial biomarkers predictive of disease progression or regression (Lavitola et al., 2020). The intricate relationship between the vaginal microbiome and HPV highlights the need for comprehensive, multi-center cohort studies to clarify these interactions and guide clinical practice. Such studies should account for confounding factors—including sexual behavior, hormonal status, and co-infections—that influence microbiota composition and HPV dynamics. Large-scale cohorts are vital for generating reliable evidence, identifying key risk modifiers, and informing targeted prevention and therapeutic strategies to improve outcomes in women at risk of HPV-related cervical disease.

### 2.9 Comprehensive overview of vaginal microecology and prevention strategies for HPV-related cervical lesions

#### 2.9.1 Comprehensive microbial regulation combined with HPV vaccination strategies

The interaction between the vaginal microbiome and HPV infection is a key focus for advancing prevention and treatment strategies. Studies, including a cross-sectional survey of young Swedish women, show that *Lactobacillus*-dominated microbiota protect against HPV and cervical lesions, while non-*Lactobacillus* dominance correlates with oncogenic HPV types (Cheng et al., 2020). These findings support integrating microbiota modulation with HPV vaccination. Probiotics and prebiotics can restore microbial balance, reduce inflammation and oxidative stress, and strengthen antiviral immunity (Huang et al., 2024). Furthermore, therapeutic HPV vaccines employing bacterial vectors may leverage microbiome-driven immune modulation to enhance vaccine efficacy and promote HPV clearance (Vargas-Robles et al., 2023).

The synergistic effects of combining microbiome regulation with HPV vaccination could be particularly beneficial in populations with high HPV prevalence and associated cervical cancer risk, such as women living with HIV (McClymont et al., 2022). Populations with altered immunity and higher susceptibility to HPV-related diseases, such as women living with HIV, require innovative approaches that integrate microbial health with vaccination. Mucosal vaccines that elicit both systemic and local immune responses, combined with probiotics that strengthen the vaginal microbiota, could provide a comprehensive, multi-layered defense against HPV infection and disease progression (Gong et al., 2023). Additionally, identifying microbial signatures linked to HPV infection may enable targeted interventions and personalized vaccination strategies tailored to high-risk populations (Fraszczak et al., 2022).

Emerging evidence indicates that vaginal microbiome composition can affect HPV vaccine efficacy, with specific bacterial taxa linked to stronger vaccine-induced immune responses (Giraldo et al., 2021). A dysbiotic microbiome may impair vaccine immunogenicity, emphasizing the need to understand microbial influences on immunity. Optimizing the vaginal microbiota before vaccination could enhance immune protection against HPV and reduce the risk of cervical cancer.

In conclusion, combining microbiome modulation with HPV vaccination offers a promising strategy to strengthen prevention and treatment of HPV-related diseases. Promoting a balanced vaginal microbiota and harnessing the immune-enhancing effects of probiotics and therapeutic vaccines may reduce HPV persistence and improve women's health globally. Future research should clarify how the microbiome shapes HPV infection and vaccine responses and develop tailored interventions that meet the needs of diverse populations.

#### 2.9.2 Early screening and individualized interventions

The link between vaginal microecological disturbances and persistent HPV infection has become a key focus in early screening and personalized intervention research. Monitoring vaginal microecology is essential for evaluating HPV infection risk and cervical lesion development (Fan et al., 2024). A *Lactobacillus-dominant* microbiome supports local immunity and protects against pathogens, whereas dysbiosis—marked by reduced *Lactobacillus* and increased pathogenic bacteria—is associated with greater HPV susceptibility and cervical cancer progression (Nittala et al., 2024).

Recent advances in genomic medicine and precision medicine have created new opportunities for early detection and personalized intervention in cervical cancer prevention. Precision Population Medicine (PPM) integrates individual risk factors—such as microbiome composition, genetic susceptibility, and environmental exposures—into tailored health strategies. Leveraging big data analytics, wearable monitoring devices, and telemedicine enables more accurate identification of women at risk for HPV infection and cervical disease, promoting timely and targeted screening and intervention (Nittala et al., 2024).

Furthermore, individualized interventions guided by vaginal microecological assessments can strengthen preventive strategies. Women with dysbiotic microbiomes may benefit from targeted probiotic therapy to restore *Lactobacillus-dominant* flora, potentially reducing HPV infection risk and enhancing cervical health. Personalized education and counseling on sexual health, HPV vaccination, and regular screening further empower women to actively manage and protect their reproductive health (Nittala et al., 2024).

Integrating microecological monitoring into routine gynecological care marks a paradigm shift in assessing and managing HPV risk. As understanding of the vaginal microbiome–HPV interplay deepens, these insights can inform more precise screening and personalized interventions. Such a proactive approach improves early detection of HPV-related cervical lesions and supports the broader goal of reducing cervical cancer incidence and mortality, especially in underserved populations (Nittala et al., 2024).

In conclusion, monitoring vaginal microecology is a vital element of early screening and personalized intervention for HPV infection and cervical disease. Integrating microbiome assessment within precision medicine frameworks can improve risk stratification, strengthen prevention, and advance efforts toward cervical cancer elimination. Continued research on the vaginal microbiome's role in HPV dynamics will refine these strategies, bringing us closer to a future where cervical cancer is no longer a major public health threat.

#### 2.9.3 Public health strategies and health education promotion

The link between vaginal microbiota, HPV infection, and cervical lesions highlights the need for public health initiatives that promote awareness of vaginal health and HPV prevention. A *Lactobacillus-dominant* microbiome protects against HPV persistence, whereas dysbiosis increases susceptibility to infection and lesion development (Kazlauskaite et al., 2025; Cui et al., 2025). Educational programs should emphasize the importance of maintaining microbial balance, which is influenced by sexual behavior, hygiene, and antibiotic use. Notably, women with BV have a significantly higher risk of persistent HPV infection and cervical lesion progression (Na et al., 2025).

Health education campaigns should stress the importance of regular gynecological check-ups, HPV vaccination, and awareness of vaginal infection symptoms. Educating women about lifestyle factors—such as smoking, which is linked to persistent HPV infection—can encourage healthier choices (Na et al., 2025). Additionally, the integration of microbiota education into routine healthcare can help women recognize the impact of their vaginal health on overall reproductive health. For example, promoting the use of probiotics and prebiotics as potential adjunct therapies to restore healthy vaginal flora could be beneficial (Papamentzelopoulou and Pitiriga, 2025).

Addressing cultural stigmas and misconceptions is essential, as discomfort in discussing vaginal health often leads to delayed diagnosis and treatment. Community outreach, educational workshops, and social media campaigns can provide accessible platforms to share information on vaginal health, HPV prevention, and the importance of maintaining microbiota balance.

Integrating these educational strategies into public health initiatives can greatly improve women's understanding of reproductive health and promote proactive care. Empowering women to take charge of their vaginal health can reduce HPV infection rates and cervical disease incidence, ultimately enhancing overall health and quality of life.

## 3 Discussion

Although this review discusses the potential “dual role” of VVC in HPV infection, the current evidence supporting these two effects is clearly imbalanced. The HPV-promoting role of VVC—mediated by inflammation, epithelial disruption, and microecological imbalance—is supported by multiple mechanistic and epidemiologic studies. In contrast, the proposed suppressive effect relies on limited cross-sectional findings and theoretical immune-activation pathways, with inconsistent results across studies. Therefore, this inhibitory effect should be interpreted as preliminary and context-dependent, whereas the available evidence more strongly supports VVC as a contributor to HPV persistence. Further longitudinal and mechanistic research is required to clarify whether VVC genuinely confers meaningful antiviral effects.

## 4 Conclusion

The strong association between persistent HPV infection and cervical disease underscores the pivotal role of high-risk HPV types in cervical carcinogenesis. This review highlights the growing recognition of the vaginal microbiome's influence on HPV dynamics, showing that microecological disruptions—particularly from BV, TV, and VVC—affect HPV infection and persistence. The dual role of VVC, which can both promote and inhibit HPV infection, further emphasizes the complexity of host–microbiota–virus interactions and the need for continued in-depth research.

Notably, disturbances in the vaginal microbiota have been linked to increased expression of HPV oncogenes E6 and E7. The detection of E6/E7 mRNA as an auxiliary diagnostic tool offers promise for improving early identification and management of high-risk HPV infections. These insights underscore the importance of understanding HPV–microbiome interactions to refine diagnostic approaches and optimize patient care..

The restoration and regulation of vaginal microecology emerge as pivotal components in the prevention and treatment of HPV infections. Integrating microbiome modulation strategies with HPV vaccination could significantly enhance the efficacy of cervical disease prevention efforts. This multifaceted approach not only addresses the viral aspect of HPV but also acknowledges the importance of the host's microenvironment in influencing disease outcomes.

Looking ahead, future research should focus on elucidating the molecular mechanisms underlying the interactions between the vaginal microbiome and HPV. Large-scale clinical studies are needed to clarify these complex relationships and guide the development of tailored interventions that account for individual microbiome variations and HPV susceptibility. These efforts could advance precision medicine in cervical cancer prevention and treatment, enabling more effective, personalized strategies for diverse patient populations.

In conclusion, the intersection of HPV infection, vaginal microbiome dynamics, and cervical disease presents a compelling area for ongoing research and clinical focus. By balancing the various research perspectives and findings, we can foster a comprehensive understanding that not only informs clinical practice but also guides public health initiatives aimed at reducing the burden of cervical cancer globally. Integrating microbiome research into HPV-related studies will be critical for informing future guidelines and advancing effective strategies against this preventable disease.
